# Internet Use, Subjective Age, and Mental Health Among Middle‐Aged and Older Adults in China: Pressured Versus Voluntary Internet Use

**DOI:** 10.1155/da/9334404

**Published:** 2026-08-26

**Authors:** Long Li, Haiyi Cheng, Guangzhao Jin, Rize Jing

**Affiliations:** ^1^ Center for Population and Development Studies, Renmin University of China, Beijing, China, ruc.edu.cn; ^2^ Beijing Higher Education Innovation Center for Population Development and Governance Research, Renmin University of China, Beijing, China, ruc.edu.cn; ^3^ School of Population and Health, Renmin University of China, Beijing, China, ruc.edu.cn; ^4^ Department of Sociology, University of Virginia, Charlottesville, Virginia, USA, virginia.edu; ^5^ School of Sociology, Nankai University, Tianjin, China, nankai.edu.cn; ^6^ Bigdata and Responsible Artificial Intelligence for National Governance, Renmin University of China, Beijing, China, ruc.edu.cn

**Keywords:** depressive symptoms, digital divide, structural equation modeling, subjective age

## Abstract

**Background:**

The rapid development of Internet technology and the swift digitization of public services may expose middle‐aged and older adults to challenges of pressured Internet use. This study aimed to compare the relationships between pressured and voluntary Internet use and mental health among Chinese middle‐aged and older adults, and to explore the mediating role of subjective age.

**Methods:**

Based on data from the China Digital Divide and Digital Inclusion Survey (CDDDIS) involving 5231 individuals aged ≥50 years, confirmatory latent class analysis (LCA) was employed to identify pressured and voluntary Internet use patterns. Structural equation modeling, adjusting for covariates, was utilized to examine their differential associations with depressive symptoms and the mediating effects of subjective age.

**Results:**

Among middle‐aged and older Internet users, 27% engaged in pressured Internet use. Voluntary Internet use demonstrated significant direct and indirect effects on reducing depressive symptoms via lowering subjective age. Pressured Internet use showed no discernible effect on depressive symptoms compared to nonuse.

**Conclusion:**

Voluntary Internet engagement enhances mental health among middle‐aged and older adults by mitigating stereotyped self‐perceptions of aging, whereas unwilling technology interactions fail to yield psychological benefits. Supporting self‐driven digital participation is crucial in addressing well‐being disparities in the context of proliferating Internet services.

## 1. Introduction

The rapid expansion of Internet technology has transformed daily life globally, with varying penetration rates across countries. By 2024, Internet users represented over 93% of individuals in high‐income countries, while low‐ and middle‐income countries (LMICs) showed lower rates of around 67% [1]. Thus, while Internet connectivity ceased to be a primary concern for the general population in most high‐income countries, its rapid diffusion persisted in LMICs. China has experienced one of the world’s most dramatic digital transformations, with Internet usage increasing from 33% to 78% between 2010 and 2024 [2]. As younger generations have largely mastered Internet technologies, middle‐aged and older adults aged 50 and above are emerging as the main growing demographic of Internet users during rapid diffusion and are experiencing the most pronounced impacts [3]. In China, this group exhibits the largest differences in Internet usage rates compared to other groups. Specifically, according to the data from the China Internet Network Information Center (CNNIC), before the COVID‐19 pandemic, while 90% of adults aged 20–49 use the Internet, usage drops to 67% for those aged 50–59 and 42% for those 60 and older [2]. At the same time, this group also shows the largest differences in Internet usage rates between China and high‐income countries. Middle‐aged and older adults often lack digital literacy and skills yet face increasing pressure to use the Internet and adopt new technologies as Internet access and digitalization continue to expand, alongside the migration of government and public services online [4], resulting in pressured Internet use. Pressured Internet use refers to the situation where individuals involuntarily resort to using certain Internet applications in certain social contexts, especially amidst sudden social circumstances related to the rapid spread of Internet technology and the accelerated digitization of public services. This phenomenon occurs when individuals involuntarily turn to the Internet, driven by the scarcity or inaccessibility of previously viable offline channels, especially when offline services are significantly replaced or entirely supplanted by online services. In such scenarios, individuals heavily rely on, or are compelled to rely on, online channels to access public resources and obtain public permissions. For example, hospitals may discontinue offline registration channels, and governmental public releases become exclusively accessible through the Internet.

During the COVID‐19 pandemic in China, daily activities such as entering public spaces (e.g., markets, hospitals, and schools) and transportation depended on health QR codes and travel tracking applications to coordinate outbreak containment efforts [5]. Consequently, many middle‐aged and older adults with limited prior technology experience turned to these mandatory platforms amidst the quarantine measures. This interplay between the sudden digitization of daily logistics and the lagging digital readiness of middle‐aged and older adults exemplified the dynamics that led to pressured Internet use. According to the CNNIC [2], by the beginning of 2020, the Internet use rate for individuals aged 60 years and above was 24%, marking an 11% cumulative increase over the three‐year period from the beginning of 2017. However, by early 2023, the Internet use rate among older adults had reached 55%, indicating a cumulative increase of 31% over the three‐year period from early 2020, and this coincides with the COVID‐19 prevalence time. This rapid surge in Internet use, with a growth rate almost three times higher than previous trends, effectively illustrates the phenomenon of pressured Internet use. While many countries, including China, have phased out pandemic control strategies reliant on digital credentials, the rapid spread of Internet technology and the accelerated digitization of public services in LMICs suggest that significant, and in some cases exclusive, access to service resources will be facilitated through the Internet. As a result, pressured Internet use is poised to persist in the foreseeable future. However, academic research on pressured Internet use remains scarce. The study investigating pressured Internet use represents a crucial extension of digital divide research, shedding light on new dimensions beyond access and basic adoption.

Mental health constitutes an important facet of well‐being for middle‐aged and older adults. Extensive studies have attempted to establish a link between Internet use and psychological well‐being among middle‐aged and older adults [6, 7]. However, results have not been entirely consistent and even fueled debates between proponents of the “techno‐evangelist” and “techno‐sceptic” approaches [8]. Part of this inconsistency can be attributed to the insufficient consideration of the heterogeneity of Internet use behaviors. Previous research has attempted to categorize Internet use behaviors into different types, such as socializing, enjoying entertainment, information seeking, and shopping, and explored the different psychological well‐being associated with these categories [7]. Within these categorized use types, however, both pressured Internet use and its counterpart, voluntary Internet use, exist. Pressured Internet use involves passive engagement, whereas voluntary Internet use involves active initiation, representing different technological identities and resulting in different self‐perceptions [9]. Given that self‐perceptions, particularly those represented by subjective age, are significantly predictive of mental health, it is expected that pressured and voluntary Internet use will exhibit different associations with mental health among middle‐aged and older adults. The amalgamation of these two dimensions has hindered our ability to elucidate the potential effects on the mental health of being involuntarily drawn into Internet activities. As Internet expansion and the digitization of public services continue in LMICs, the specific impact of pressured Internet use on psychological well‐being emerges as a crucial issue requiring research attention.

### 1.1. Pressured Versus Voluntary Internet Use

Drawing on Barton’s [10] concept of property space, Blank and Groselj [11] proposed a framework for analyzing Internet use patterns across three dimensions: amount, variety, and type of use. Within this framework, pressured Internet use typically involves narrow, frequent engagement with specific applications tied to particular social contexts, with minimal exploration of other online activities. In contrast, voluntary Internet use encompasses diverse behaviors driven by personal preferences rather than external constraints. The difference between pressured and voluntary Internet use extends beyond usage patterns to reflect distinctive technological identities [9]. Martin’s [12] theoretical framework identifies four dimensions of technological identity: beliefs about technology’s importance, perceptions of one’s technological abilities, beliefs about participation opportunities/constraints, and motivation to learn more. Compared to voluntary users, pressured Internet users typically exhibit greater skepticism about their technological abilities and participation opportunities, indicating a less fluent and more challenged technological identity. Although both groups recognize the importance of technology, the motivation to learn more about technology is significantly lower among pressured Internet users. This technological identity will continue to shape their self‐perception through their daily interactions with technology and influence their self‐concept. These contrasting technological identities may influence users’ self‐perceptions and psychological outcomes.

Within the theme of the digital divide, scholars have extensively examined differences in Internet use types based on differences in attitudes, approaches to use, skill levels, and frequency of Internet use (see reviews by Fang et al. [4]). The varied consequences of different Internet use types have also been explored [13, 14]. However, as voluntary Internet use may exhibit preferences for different types of Internet use, and it remains challenging to effectively collect information on Internet readiness in traditional surveys and link it to specific social contexts, pressured Internet use is relatively difficult to identify easily based on the observed type and frequency of Internet use behaviors in practice. The COVID‐19 containment efforts in China provided a unique opportunity for research. During the pandemic, national authorities introduced a travel code system, while local governments implemented specialized health QR code systems, such as the Health Kit (*Jiankangbao*) in Beijing [5]. These applications, which compiled data on the travel history, nucleic acid test results, and vaccination status of individuals, served as critical tools for accessing many offline spaces (e.g., public transport, markets, hospitals, schools, etc.). The presentation of travel and health codes was necessitated to comply with pandemic control requirements for public access and transit or to secure release from quarantine. Consequently, these applications became indispensable in daily life, with the absence of digital credentials causing significant inconvenience. As a result, middle‐aged and older adults who had previously not engaged with the Internet were compelled to adopt and frequently utilize it. By identifying behaviors exclusively associated with frequent use of applications related to pandemic prevention and control, we can more accurately determine pressured Internet use. In contrast, voluntary Internet use encompasses the other Internet use behaviors beyond this pressured behavior.

### 1.2. Pressured and Voluntary Internet Use and Mental Health

Over recent decades, the focus of digital divide research has shifted from examining mere access to the Internet (the first‐level digital divide) to investigating how individuals use the Internet (the second level), involving skills necessary for digital device use, differential digital practices, and specific Internet uses. There is also an increasing emphasis on understanding the differential offline outcomes that individuals obtain from their use of digital technologies, known as the third‐level digital divide [15, 16]. Numerous empirical studies have highlighted the mental health consequences of Internet use among middle‐aged and older adults [17–19]. Depression, in particular, has been scrutinized as an indicator of mental health in such studies, and Internet use among middle‐aged and older adults is closely associated with depression across several dimensions, including access, diversity of use, and specific types. Overall, online participation is correlated with lower levels of depression among middle‐aged and older adults [20, 21]. However, the majority of existing research has been conducted in settings where Internet use is predominantly voluntary and self‐initiated, primarily in high‐income countries [22, 23]. The prevalence of pressured Internet use amid the rapid diffusion of Internet technologies and the accelerated digitization of public services in today’s LMICs has received limited attention. The relationship between pressured and voluntary Internet use (a typical manifestation of the second‐level digital divide) and the mental health among middle‐aged and older adults remains largely unknown within the context of the third‐level digital divide.

The uses and gratifications theory provides a valuable framework for understanding Internet use and its psychological effects among older adults [24, 25]. This theory posits that individuals engage with media based on expected benefits, which shape usage patterns and outcomes [26, 27]. Integration with social cognitive theory suggests that these expectations relate to technological identity, which influences orientation toward digital technology, internalization tendencies, attitudes, and emotional experiences [28, 29]. In voluntary Internet use, proactive technological exploration aligns with a fluent technological identity, fostering enthusiasm and positive expectations that contribute to favorable mental health outcomes [30]. Conversely, individuals lacking digital fluency—particularly in pressured use scenarios—often experience what Lave and Wenger [31] describe as “disruptive peripheral participation.” They encounter preconfigured applications that may not match their utility needs, leading to frustration and potential negative mental health consequences [32, 33]. Furthermore, use satisfaction derives from three primary sources: process, content, and social gratifications [25]. Pressured Internet use typically yields primarily process satisfaction through superficial engagement with required applications. This limited engagement generally provides less psychological benefit than the more comprehensive satisfaction (process, content, and social) associated with voluntary use [6, 7]. Based on these observations, this study proposes the following research hypotheses:

Hypothesis 1: Pressured and voluntary Internet use exhibit differential associations with mental health among middle‐aged and older adults.
Hypothesis 1a: Compared to nonuse, pressured Internet use will not show a significant positive association with mental health among middle‐aged and older adults.
Hypothesis 1b: Compared to nonuse, voluntary Internet use will show a significant positive association with mental health among middle‐aged and older adults.

### 1.3. The Mediating Role of Subjective Age

Subjective age, the subjective experience of one’s own aging among middle‐aged and older adults, is a crucial aspect of their self‐perception [34]. Existing research generally highlights subjective age as a key predictor of the psychological well‐being among middle‐aged and older adults [35, 36]. A lower subjective age, indicative of a positive outlook on the aging process, is typically associated with higher levels of subjective well‐being, more positive emotional experiences, and reduced risk of depression [35, 37–39]. Theoretical explanations often attribute these associations to the internalization of age stereotypes and self‐fulfilling prophecies. The perceived threat of age‐related stereotypes among middle‐aged and older adults can evoke direct stress responses [40, 41]. These age stereotypes become self‐relevant under the influence of societal and cultural factors, leading to internalized beliefs that manifest as self‐ageism. Subsequently, such internalized beliefs negatively impact the psychological well‐being of middle‐aged and older individuals through psychological pathways. Conversely, middle‐aged and older adults may also engage in social comparisons with peers and assimilate themselves with younger individuals [42] to achieve a younger subjective age. This process serves as a protective mechanism against the negative effects of age stereotype threat, ultimately promoting psychological well‐being and reducing the tendency towards depression [43].

Integrating the uses and gratifications theory with social cognitive theory, the process of use gratification involves a vital self‐regulatory process for self‐reflective media like the Internet, and this primarily involves the judgmental process based on self‐monitoring of one’s behavior in accordance with personal and societal norms, as well as self‐reaction [24]. In today’s highly digitized context, voluntary Internet use implies a strong alignment between technological identities and mainstream norms (youth norms) compared to nonuse. Through peer comparison, middle‐aged and older adults actively engaging with the Internet mitigate ageism and achieve a subjectively younger age, thereby reducing the threat of age stereotypes and the negative impact of self‐ageism on their well‐being. Conversely, pressured Internet use, characterized by disruptive peripheral participation [31], elicits anxiety regarding the capabilities of middle‐aged and older adults [24] and propagates stereotype‐consistent technology identities that contribute to feelings of comparative older age and negative perception of aging [44]. Self‐disparagement and self‐slighting may afflict pressured Internet users who compare their abilities to standards set by the most accomplished Web users, like the youth, and by constantly changing Internet technology, which reduces the self‐reaction incentive. In addition, research indicates associations between limited technology use and greater subjective age, as well as more negative self‐views on aging [45–47]. Accordingly, increased digital engagement is associated with a subjective sense of feeling younger and more positive about one’s aging process. These findings provide critical empirical support for this study. By elucidating these mechanisms underlying Internet use and subjective age, the current study advances discourses on digital inequality and aging alike. Based on these considerations, this article proposes the following research hypotheses:

Hypothesis 2: Pressured and voluntary Internet use exhibit differential associations with subjective age, which is related to mental health among middle‐aged and older adults.
Hypothesis 2a: Compared to nonuse, pressured Internet use is expected to increase the subjective age, contributing to a worsening of mental health among middle‐aged and older adults.
Hypothesis 2b: Compared to nonuse, voluntary Internet use is expected to lower the subjective age, contributing to an amelioration of the mental health of middle‐aged and older adults.

### 1.4. The Present Study

This study aims to develop more precise identification approaches for pressured versus voluntary Internet use in the Chinese context, examine their respective relationships with mental health among middle‐aged and older adults, and explore subjective age as a mediating mechanism. By distinguishing between these use patterns, we contribute to understanding the psychological implications of differing technological engagement pathways during rapid digital transformation.

## 2. Material and Methods

### 2.1. Sample and Data

Data were sourced from the China Digital Divide and Digital Inclusion Survey (CDDDIS) conducted by the Center for Population and Development Studies at Renmin University of China (November 2022–January 2023). The CDDDIS employed a stratified multistage probability‐proportional‐to‐size sampling method across 18 provinces in China, covering 53 counties/districts and 252 rural villages/urban communities. The survey involved exclusively face‐to‐face interviews with participants aged 50 and above, with an intentional oversampling of Internet users to facilitate robust analysis of digital behaviors. A total of 5231 participants, comprising 4293 Internet users and 938 nonusers, were included in this study. The study was not preregistered. All procedures involving human participants were reviewed and approved by the Ethics Committee of Renmin University of China. Written informed consent was obtained before each interview. Interviewers first introduced themselves and provided a verbal explanation of the study’s purpose, procedures, potential risks and benefits, voluntary participation, and confidentiality protections. Participants were then given a written consent form summarizing this information, with sufficient time for questions before signing.

To ensure confidentiality and data protection, comprehensive anonymization and data management procedures were implemented. Responses were collected using structured questionnaires administered in person, thereby minimizing digital security risks during data capture. All data were deidentified immediately after collection through the use of unique study identifiers in place of personal information. The analytic sample (*N* = 5231) was reported in aggregated form only, and no individual‐level information was disclosed. Data were stored in encrypted files accessible solely to the authorized research personnel and were used exclusively for scientific purposes. As noted in the Data Availability Statement, the dataset is available from the authors upon reasonable request to further safeguard against unauthorized access. Protocols for managing potential participant distress—including indications of suicidal ideation or acute anxiety—were also established, with interviewers trained to follow predefined referral and escalation procedures when necessary.

### 2.2. Measures

#### 2.2.1. Pressured and Voluntary Internet Use

We employed confirmatory latent class analysis (LCA) to identify patterns of pressured and voluntary Internet use. LCA is increasingly used to characterize unobserved subgroups based on responses to categorical variables [48–50]. Participants reported their ability to perform various online activities (yes = 1, no = 0) including: checking health QR codes, information seeking, shopping, payment, transportation services, health management, financial activities, messaging, entertainment, news consumption, gaming, and e‐learning.

As mentioned previously, pressured and voluntary Internet users have common response probabilities for using health QR codes but divergent probabilities for other online activities. According to Finch and Bronk [50], in the two‐class LCA model, we adjusted the variable threshold used in *Mplus* 7.4 for the item “checking and scanning health QR codes” to ensure high endorsement probabilities in both classes. For other items (e.g., “information seeking”), we set the threshold for the latent pressured Internet users to be the negative of those for the latent voluntary Internet users, indicating that the latent pressured Internet users have a lower probability of endorsing these items than the latent voluntary Internet users. Additionally, nonusers were included as a comparison group.

We also tested a series of unconstrained LCA models from one‐class to five‐class and adopted an array of fit indices to evaluate the performance of the two‐class LCA model. The fit indices included the Akaike information criterion (AIC), Bayesian information criterion (BIC), sample‐size adjusted BIC (aBIC), and entropy, with the lower values of the first three indices and the higher value of entropy indicating a better fit of the model [48]. However, the AIC, BIC, and/or aBIC values may never reach the lowest value with the increase in the number of classes. In such scenarios, the class at the inflection point may be considered the most suitable [51–53]. The Lo–Mendell–Rubin (LMR) test and the bootstrap likelihood ratio (BLR) test are also utilized to assess model fit, with statistically significant results suggesting a good fit. As shown in Figure S1, among the unconstrained LCA models, the inflection point occurred at the two‐class solution, which also reported the highest entropy. The fit indices for the two‐class confirmatory LCA solution were equal to those of the two‐class unconstrained solution. Both the LMR test and the BLR test of the two‐class confirmatory LCA yielded statistically significant results (*p* < 0.001). Therefore, the two‐class confirmatory LCA model fits the data.

#### 2.2.2. Depressive Symptoms

Mental health was assessed using the 10‐item Center for Epidemiologic Studies‐Depression (CESD) scale [54, 55], which evaluates negative/positive emotions and somatic symptoms experienced over the past week. Responses used a four‐point Likert scale (1 = rarely/none of the time to 4 = most/all of the time). After reverse‐coding positive items, scores were summed (range: 10–40), with higher scores indicating a greater depression risk. The scale demonstrated acceptable reliability (Cronbach’s *α* = 0.71).

#### 2.2.3. Subjective Age

Following established approaches [34, 56, 57], subjective age was measured by asking participants “How old do you feel most of the time?” (range: 0–100). We calculated proportional difference scores by subtracting the chronological age from the subjective age and dividing by the chronological age. Negative scores indicated feeling younger than one’s chronological age, while positive scores indicated feeling older.

#### 2.2.4. Covariates

Based on prior research [55, 58], we included sociodemographic, socioeconomic, and health‐related covariates: age, sex (female/male), marital status (married/other status), household size, years of education, employment status (nonworking/currently working), rural/urban status (rural/urban), and functional health assessed by activities of daily living (ADL) score.

### 2.3. Analytic Strategy

Descriptive analyses were conducted for all variables of interest, presenting the mean (standard deviation [SD]) or percentage. Descriptive statistics included the characteristics of the total sample and the subgroups of pressured and voluntary Internet users, along with nonusers. Path analysis was employed to examine potential variations in the associations between pressured and voluntary Internet use and depressive symptoms (Hypothesis 1) and to identify the potential mediating effects of subjective age on these associations (Hypothesis 2) among middle‐aged and older adults in China. All covariates were adjusted for in each model. Model fit was assessed using established goodness‐of‐fit indices, including the comparative fit index (CFI), root mean square error of approximation (RMSEA), and standardized root mean square residual (SRMR). A CFI value exceeding 0.90, a RMSEA value below 0.06, and a SRMR value below 0.08 were considered indicative of satisfactory model fit for this study [59]. All of the above analyses were conducted using *Mplus* 7.4 [60] and Stata 16.0 [61].

## 3. Results

### 3.1. LCA Model Validation

Response probabilities for Internet activities showed clear differentiation between user types (Table 1). Both pressured and voluntary users showed high probabilities for health QR code usage (>0.80), reflecting pandemic requirements. However, for all other activities, voluntary users demonstrated substantially higher engagement probabilities, particularly for information seeking (0.74 vs. 0.31), online shopping (0.85 vs. 0.22), and payment (0.94 vs. 0.66).

**Table 1:** Item‐response probabilities in two‐class model of pressured and voluntary Internet use patterns.

Internet activities	Pressured Internet use	Voluntary Internet use
Checking and scanning health QR codes	0.829	0.961
Information seeking	0.307	0.735
Shopping	0.218	0.848
Payment	0.660	0.936
Transportation	0.051	0.554
Managing health	0.035	0.316
Investment and financing	0.009	0.220
Instant messaging	0.943	0.993
Entertainment	0.660	0.973
Watching or listening to news	0.371	0.696
Playing games	0.088	0.426
E‐learning	0.130	0.506
*N*	1172	3121

### 3.2. Sample Characteristics

Table 2 presents the descriptive statistics for all analytic variables. The mean age of participants (*N* = 5,231) was 62.09 years (SD = 9.42), with 51% being female. Most participants (86%) were married, with an average household size of 2.97 people (SD = 1.46). The mean education level was 9.01 years (SD = 3.60), with 42% employed and 53% residing in urban areas. Overall, participants showed a mean depressive symptom score of 17.81 (SD = 4.13) and a subjective age of −0.06 (SD = 0.09), indicating that they felt ~6% younger than their chronological age.

**Table 2:** Descriptive statistics of the sample.

Variables	Total (*N* = 5231)	Pressured Internet users (*N* = 1172)	Voluntary Internet users (*N* = 3121)	Nonusers (*N* = 938)
Age, mean (SD)	62.09 (9.42)	63.13 (7.90)	57.53 (5.89)	75.98 (6.19)
Sex, *N* (%)				
Female	2671 (51%)	615 (52%)	1540 (49%)	516 (55%)
Male	2560 (49%)	557 (48%)	1581 (51%)	422 (45%)
Marital status, *N* (%)				
Married	4521 (86%)	1012 (86%)	2946 (94%)	563 (60%)
Other status	710 (14%)	160 (14%)	175 (6%)	375 (40%)
Household size, mean (SD)	2.97 (1.46)	2.98 (1.65)	2.97 (1.36)	2.93 (1.52)
Years of education, mean (SD)	9.01 (3.60)	8.40 (3.35)	10.27 (2.84)	5.61 (3.78)
Employment status, *N* (%)				
Nonworking	3059 (58%)	818 (70%)	1428 (46%)	813 (87%)
Currently working	2172 (42%)	354 (30%)	1693 (54%)	125 (13%)
Rural/urban status, *N* (%)				
Rural	2464 (47%)	631 (54%)	1388 (44%)	445 (47%)
Urban	2767 (53%)	541 (46%)	1733 (56%)	493 (53%)
ADL, mean (SD)	20.63 (1.12)	20.69 (0.97)	20.79 (0.74)	20.03 (1.87)
Depressive symptoms, mean (SD)	17.81 (4.13)	17.93 (4.32)	17.37 (3.85)	19.14 (4.50)
Subjective age, mean (SD)	−0.06 (0.09)	−0.05 (0.08)	−0.07 (0.10)	−0.02 (0.06)

Among Internet users, 73% were classified as voluntary users, while 27% exhibited pressured use patterns. Pressured Internet users tended to be older, with two‐thirds being aged over 60. They also reported lower educational attainment, higher rates of retirement/inactivity, and predominantly rural residence. Regarding mental health, pressured Internet users reported higher depressive symptoms compared to voluntary users (17.93 vs. 17.37). In terms of subjective age, pressured Internet users perceived themselves as 5% younger than their chronological age, while voluntary users felt 7% younger. Nonusers were characterized by the oldest age profile, lowest educational attainment, and highest rates of labor market disengagement.

### 3.3. Path Models for Internet Use Patterns, Subjective Age, and Depressive Symptoms

**Figure 1:**
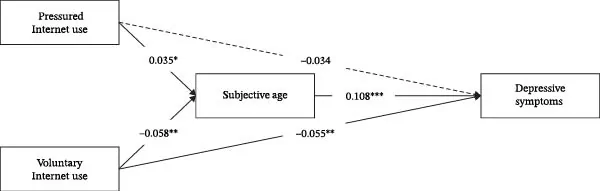
Path coefficients for the model regarding pressured and voluntary Internet use, subjective age, and depressive symptoms (*N* = 5231). All the path coefficients are standardized and adjusted for age, sex, marital status, household size, years of education, employment status, rural/urban status, and ADL. Solid lines indicate statistically significant paths (*p* < 0.1), whereas dotted lines indicate nonsignificant paths.  ^∗∗∗^ *p* < 0.01;  ^∗∗^ *p* < 0.05;  ^∗^ *p* < 0.1.

Figure 1 displays estimates for the main paths among Internet use patterns, subjective age, and depressive symptoms after adjusting for covariates. The result of path analysis demonstrated excellent fit: CFI = 0.99, RMSEA < 0.01, SRMR < 0.01. Voluntary Internet use showed a significant direct negative association with depressive symptoms (*β* = −0.055, *p* = 0.038), while pressured Internet use showed no significant direct association with depressive symptoms. This finding supports Hypotheses 1a and 1 b, indicating differential mental health outcomes across Internet use patterns.

Regarding potential mechanisms, voluntary Internet users reported significantly lower subjective age (*β* = −0.058, *p* = 0.030), which was negatively associated with depressive symptoms. Conversely, pressured Internet use showed a marginally positive association with subjective age (*β* = 0.035, *p* = 0.099), potentially contributing to higher depression risk, though this effect did not reach conventional significance thresholds.

Table 3 presents the direct, indirect, and total effects from the mediation models. The total effect from voluntary Internet use to depressive symptoms was statistically significant (*β* = −0.514, *p* = 0.021). The indirect effect via subjective age was also significant (*β* = −0.052, *p* = 0.037), accounting for ~10.2% of the total effect. However, no significant direct, indirect, or total effects were observed between pressured Internet use and depressive symptoms, further supporting our hypotheses regarding differential associations with mental health.

**Table 3:** Estimate for the direct and indirect effects of pressured and voluntary Internet use via subjective age on depressive symptoms.

Pathways	Direct effect	Indirect effect	Total effect
Pressured Internet use → depressive symptoms	−0.332	0.037	−0.295
Voluntary Internet use → depressive symptoms	−0.462 ^∗∗^	−0.052 ^∗∗^	−0.514 ^∗∗^

^∗∗^ *p* < 0.05.

### 3.4. Additional Analyses

We conducted additional sensitivity analyses. In this study, 46.7% of the participants were younger than 60 years, with few pressured Internet users or even nonusers in this subgroup. Therefore, we fitted the same model in a subsample comprising only older adults aged 60 years and older to investigate whether participants’ age affects the relationships between pressured and voluntary Internet use and depressive symptoms. Based on an acceptable model fit for the data (CFI = 0.99, RMSEA < 0.01, SRMR < 0.01), this study revealed that voluntary Internet use showed a significant indirect effect on depressive symptoms through subjective age, along with a significant total effect, whereas pressured Internet use was not significantly related to depressive symptoms among older adults. These results are presented in Table S1 and Figure S2.

We conducted the second sensitivity analysis that explicitly addresses the sample‐size imbalance between different Internet‐use groups. We construct group‐specific weights to equalize the effective sample sizes of pressured and voluntary Internet users. The weighted results are reported in Table S2. As shown there, the estimated differences in CES‐D across the three Internet‐use categories and the pattern of statistical significance are highly consistent with the unweighted models.

## 4. Discussion

In the context of China, which is representative of LMICs, the rapid proliferation of Internet technology and the swift digitization of public services may expose middle‐aged and older adults to the challenges of pressured Internet use. Despite this, scant research has delved into the dichotomy between pressured and voluntary Internet use. This study addresses this gap by exploring the application of Internet technology within the specific social context of epidemic prevention and control in China. Utilizing nationally representative data and confirmatory LCA, the study identifies distinct patterns of pressured and voluntary Internet use. Subsequently, it examines the relationships between these two novel dimensions of Internet use, situated at the second level of the digital divide, and the mental health of middle‐aged and older adults, a typical indicator of well‐being. The findings reveal that pressured Internet use is prevalent among middle‐aged and older Internet users, accounting for a significant proportion (27%). Notably, two‐thirds of pressured Internet users are aged 60 years and above. Voluntary Internet use exhibits a significant association with lower levels of depressive symptoms, while pressured Internet use shows no significant correlation with depressive symptoms. Pressured Internet use and nonuse demonstrate minimal difference in this regard. Subjective age emerges as a mediating factor, positively predicting depressive symptoms. Specifically, voluntary Internet use is significantly negatively correlated with subjective age, while pressured Internet use exhibits a positive correlation to some extent.

This study confirms differential mental health outcomes between pressured and voluntary Internet use. Voluntary Internet use demonstrated significant negative associations with depressive symptoms, aligning with previous research conducted in more autonomous settings in high‐income countries [6, 62, 63]. This confirms Hypothesis 1b. In contrast, pressured Internet use showed no significant mental health benefits compared to nonuse, supporting our Hypothesis 1a that involuntary digital engagement fails to yield the positive psychological outcomes often associated with Internet use.

This pattern of results extends our understanding of the digital divide and its consequences. While expanding Internet access and encouraging adoption are often viewed as mechanisms to reduce social inequality [64], our findings suggest that not all forms of Internet engagement yield equivalent benefits. The simple binary of “users versus non‐users” [65, 66] masks an important variation in how individuals experience and benefit from technology. The manner in which adults engage with the Internet—whether through self‐initiated exploration or external pressure—appears crucial in determining the psychological outcomes.

In addition, our study identifies subjective age as a significant mediating mechanism linking Internet use patterns and mental health, supporting Hypothesis 2. Voluntary Internet users felt subjectively younger than their chronological age, and this younger subjective age was associated with fewer depressive symptoms. This finding aligns with previous research showing that technology engagement can influence age perceptions [67, 68] and that subjective age is a robust predictor of psychological well‐being [35].

The mediating role of subjective age may reflect several underlying processes. Voluntary Internet users might experience increased self‐efficacy and autonomy through mastering new technologies, countering negative age stereotypes through successful digital engagement. Additionally, digital participation may facilitate greater social connection and cultural involvement, providing older adults with opportunities to engage with mainstream (often youth‐oriented) culture [9]. Conversely, pressured Internet users—who reported feeling subjectively older than voluntary users—may experience technology as further evidence of age‐related limitations, reinforcing rather than challenging negative age stereotypes.

These findings have important implications for digital inclusion initiatives. Programs aimed at increasing Internet use among older adults often focus primarily on providing access and basic skill training. While these components are necessary, our results suggest that they may be insufficient to promote well‐being if adoption remains externally driven rather than internally motivated. Effective digital inclusion requires not just technical training but also fostering positive attitudes toward technology and supporting autonomous exploration.

Additionally, our findings highlight the need for age‐inclusive design in digital technologies. Applications intended for public use—particularly those delivering essential services—should be developed with diverse users in mind, including those with limited digital experience. Simpler interfaces, clear navigation, and robust offline alternatives could reduce the negative experiences of pressured users and potentially transform involuntary use into more positive engagement over time.

Several limitations should be considered when interpreting our results. Our cross‐sectional design prevents establishing causal relationships among Internet use patterns, subjective age, and mental health. Longitudinal research would provide stronger evidence regarding temporal ordering and potential reciprocal effects. Additionally, our investigation of pressured Internet use focused primarily on pandemic‐related applications, potentially overlooking other common contexts of involuntary technology use, such as healthcare, transportation, and financial services. Future studies should examine pressured Internet use across diverse domains to develop a more comprehensive understanding of this phenomenon. Finally, although prior research has identified multiple psychosocial mechanisms linking Internet use to mental health—such as social adaptation and social connectedness—we focused exclusively on subjective age as the mediating variable. This choice was theoretically integrated by the uses and gratifications theory and social cognitive theory, which emphasize subjective age as an integrative indicator of individuals’ internalized aging experience, capturing both psychological and social dimensions. Nevertheless, alternative mediators were not simultaneously examined in this study, and future research could adopt a multiple‐mediator framework to compare the relative and joint explanatory roles of subjective age, social adaptation, social connectedness, and related constructs.

Despite these limitations, this study makes important contributions to our understanding of digital technology’s psychological impact on middle‐aged and older adults. By distinguishing between pressured and voluntary Internet use and identifying subjective age as a mediating factor, we provide new insights into the complex relationship between technology engagement and mental health in an aging population.

## 5. Conclusions

In this study’s context, voluntary Internet engagement enhances mental health among middle‐aged and older adults by mitigating stereotyped self‐perceptions of aging, whereas unwilling technology interactions fail to yield psychological benefits. It is of utmost importance to eschew haphazard, one‐size‐fits‐all approaches to the widespread integration of Internet technology. Such an approach may not have a significant beneficial effect on the well‐being of older adults using the Internet while also failing to reduce digital inequalities. This study indicated that supporting self‐driven digital participation is crucial in addressing well‐being disparities in the context of proliferating Internet services.

## Funding

This work was supported by grant from the Beijing Municipal Social Science Foundation (Grant 23SRC025).

## Disclosure

An earlier version of this work was presented by Long Li (first author) at the 2024 Health Sociology Graduate Forum hosted by the School of Sociology, Beijing Normal University. This presentation was for scholarly exchange purposes only and did not constitute a formal publication. The study sponsor had no role in study design, data analysis and interpretation of data, the writing of manuscript, or the decision to submit the manuscript for publication.

## Ethics Statement

This study involving human participants was reviewed and approved by Renmin University of China. The study was approved by the Ethics Committee of Renmin University of China. Participants provided their written informed consent to participate in this study.

## Conflicts of Interest

The authors declare no conflicts of interest.

## Supporting Information

Additional supporting information can be found online in the Supporting Information section.

## Supporting information


**Supporting Information** Additional supporting information is provided to present further details of the model evaluation and additional analyses. Figure S1: Presents the model fit indices for different class solutions, which supports the selection of the optimal latent class model. Tables S1 and S2: Provide estimates of the direct and indirect effects of pressured and voluntary Internet use on depressive symptoms through subjective age, including analyses among older adults and using group‐specific weighting approaches, respectively. Figure S2: Illustrates the standardized path coefficients of the structural model examining the associations among pressured Internet use, voluntary Internet use, subjective age, and depressive symptoms among older adults. *Note*: All the path coefficients are standardized and adjusted for age, sex, marital status, household size, years of education, employment status, rural/urban status, and ADL. Solid lines indicate statistically significant paths, whereas dotted lines indicate nonsignificant paths.  ^∗∗∗^ *p* < 0.01.

## Data Availability

The datasets used and/or analyzed during the current study are available from the first author and corresponding author upon reasonable request.
