# Supplementary material for: Internet Use, Subjective Age, and Mental Health Among Middle‐Aged and Older Adults in China: Pressured Versus Voluntary Internet Use
Source: Depress Anxiety. 2026 Aug 26;2026:9334404. doi: 10.1155/da/9334404 (PMC13507829; doi:10.1155/da/9334404)
Supplement: Supplementary file 1 [file DA-2026-9334404-s001.docx]

**Supplementary materials**

Figure *S*1. The Model Fits among Different Class Solutions

AIC, BIC, aBIC

Entropy

|  | Entropy |
| --- | --- |
| 1-Class | 0 |
| 2-Class | 0.738 |
| 3-Class | 0.688 |
| 4-Class | 0.68 |
| 5-Class | 0.693 |
| 2-Class | 0.738 |

| Class solutions | AIC | BIC | aBIC |
| --- | --- | --- | --- |
| LCA 1-Class | 51593.5 | 51669.8 | 51631.7 |
| LCA 2-Class | 48338.9 | 48498.1 | 48418.6 |
| LCA 3-Class | 47471.9 | 47713.8 | 47593.1 |
| LCA 4-Class | 46920 | 47244.6 | 47082.5 |
| LCA 5-Class | 46589.6 | 46996.9 | 46793.5 |
| CLCA 2-Class | 48338.9 | 48498.1 | 48418.6 |

Table S1. Estimate for the Direct and Indirect Effects of Pressured and Voluntary Internet Use via Subjective Age on Depressive Symptoms among Older Adults Aged over 60 Years

|  | Direct effect | Indirect effect | Total effect |
| --- | --- | --- | --- |
| Pressured Internet use → Depressive symptoms | -0.099 | -0.015 | -0.114 |
| Voluntary Internet use → Depressive symptoms | -0.363 | -0.130*** | -0.493* |

*Notes*: *** *p* < 0.01; * *p* < 0.1.

Table S2. Estimate for the Direct and Indirect Effects of Pressured and Voluntary Internet Use via Subjective Age on Depressive Symptoms under Group-specific Weighting

|  | Direct effect | Indirect effect | Total effect |
| --- | --- | --- | --- |
| Pressured Internet use → Depressive symptoms | -0.354 | 0.032* | -0.322 |
| Voluntary Internet use → Depressive symptoms | -0.491** | -0.059** | -0.551** |

*Notes*: *** *p* < 0.01; ** *p* < 0.05; * *p* < 0.1.

Figure S2. Path coefficients for the model regarding pressured and voluntary Internet use, subjective age, and depressive symptoms among Older Adults Aged over 60 Years (n = 2787)

*Notes*: All the path coefficients are standardized and adjusted for age, sex, marital status, household size, years of education, employment status, rural/urban status, and ADL. Solid lines indicate statistically significant paths, whereas dotted lines indicate non-significant paths. *** *p* < 0.01.
